# Multiple Myeloma and Kidney Impairment at Diagnosis: A Nephrological Perspective from an Eastern European Country

**DOI:** 10.3390/medicina59071326

**Published:** 2023-07-18

**Authors:** Gabriel Ștefan, Simona Cinca, Corina Chiriac, Adrian Zugravu, Simona Stancu

**Affiliations:** 1 Faculty of Medicine, “Carol Davila” University of Medcine and Pharmacy, 050474 Bucharest, Romaniasimonastancu2003@yahoo.com (S.S.); 2 Nephrology Department, “Dr. Carol Davila” Teaching Hospital of Nephrology, 010731 Bucharest, Romania

**Keywords:** multiple myeloma, myeloma-related kidney injury, overall survival, cast nephropathy, end-stage kidney disease

## Abstract

*Background and Objectives*: The clinical presentation and survival factors in patients with myeloma-related kidney impairment (MRKI) at diagnosis remain a topic of ongoing research, given the complex interplay between nephrology and hematology. To date, no studies have specifically reported outcomes for these patients in Eastern Europe. *Materials and Methods*: We conducted a retrospective, unicentric study of consecutive newly diagnosed patients with MRKI in our tertiary nephrology service in Romania between 2015 and 2020; follow-up extended until 1 September 2022, covering a study period of 90 months. *Results*: We identified 89 consecutive patients with MRKI (median age 66 years, 38% male, median eGFR 5 mL/min). The majority of patients had arterial hypertension (71%) and systemic atherosclerosis (58%), and the most frequent clinical features at presentation were asthenia (75%) and bone pain (51%). Light-chain-restricted myeloma was the most common type (55%), with kappa free light chain being more frequent (53%). Among the patients, 81% presented with acute kidney injury (AKI), and 38% required hemodialysis at diagnosis. During the study period, 65% of the patients died, and hypoalbuminemia and the need for hemodialysis at diagnosis were significantly associated with mortality in multivariate analysis. *Conclusions*: Patients with MRKI who present to the nephrologist more frequently exhibit light chain restriction and most often present with AKI, with one-third requiring hemodialysis at diagnosis. Moreover, hypoalbuminemia and the initiation of hemodialysis at diagnosis were significantly associated with increased mortality.

## 1. Introduction

Multiple myeloma (MM) is a hematologic malignancy characterized by the clonal proliferation of plasma cells in the bone marrow and the production of monoclonal immunoglobulin, also known as M protein [1]. This leads to various clinical manifestations, such as anemia, lytic bone lesions, hypercalcemia, and kidney impairment. Among these complications, renal dysfunction is particularly challenging, as it significantly impacts survival and quality of life [2,3].

The prevalence of kidney impairment in newly diagnosed MM patients remains unclear and largely dependent on the criteria used for definition, with rates ranging from 10% to 30% [1,3]. Kidney dysfunction in MM patients can result from various mechanisms, including monoclonal-immunoglobulin-related factors, such as cast nephropathy, light chain deposition disease, and amyloidosis, as well as factors unrelated to monoclonal immunoglobulin, including hypercalcemia, dehydration, nephrotoxic medications, infection, and pre-existing chronic kidney disease [1].

The clinical presentation and factors influencing survival in patients with myeloma-related kidney impairment continue to be debated, as this pathology encompasses a spectrum of renal dysfunction and lies at the intersection of nephrology and hematology.

Thus, diagnostic and therapeutic approaches to MM vary across Europe, with distinct challenges encountered in Eastern Europe due to differences in healthcare systems and economic parameters. In a study conducted in Central and Eastern Europe in 2018, MM patients presented somewhat less symptomatic disease as per certain CRAB criteria compared to other regions: there was a lower percentage of hypercalcemia (7% versus 13%) and renal impairment (12% versus 18–20%), but a similar percentage of patients with anemia (51% versus 45–73%) and bone pain (56% versus 58%) [4]. Moreover, there are no studies to date specifically reporting the outcomes of patients with myeloma-related kidney impairment in Eastern Europe.

The objective of our study was to provide a nephrologist’s perspective on patients with myeloma-related kidney impairment at the time of their initial diagnosis. Specifically, we aimed to evaluate the clinical presentation of these patients and identify factors related to survival during their first admission to a tertiary nephrology center in Romania.

## 2. Materials and Methods

### 2.1. Study Design and Population

We conducted a unicentric retrospective study on consecutive newly diagnosed patients with MM in our service between 2015 and 2020. Patients were followed until 1 September 2022. The study period covered a duration of 90 months (i.e., 7 years and 6 months) between the earliest admission date and the date of the retrospective cohort assessment.

Patients were included in the study if they met the following criteria: (1) a confirmed diagnosis of multiple myeloma according to the International Myeloma Working Group (IMWG) criteria [2]; (2) evidence of kidney injury at diagnosis, as indicated by any of the following: eGFR < 40 mL/min, proteinuria (urine protein-to-creatinine ratio >200 mg/g), or other laboratory or clinical evidence of kidney damage related to MM [3]; and (3) ≥18 years of age. Patients were excluded if they had a prior history of other hematologic malignancies or lacked sufficient data for analysis.

### 2.2. Data Collection

Data were extracted from electronic medical records and included demographic information, comorbidities (arterial hypertension, diabetes mellitus, ischemic heart disease, systemic atherosclerosis), clinical presentation, MM characteristics (type, free light chain level, ratio, serum electrophoresis, marrow plasmacytosis, hemoglobin level, lytic bone lesions), renal characteristics (eGFR, proteinuria, albuminuria, Bence Jones protein), additional lab tests (total serum protein, albumin, total/ionic serum calcium, lactate dehydrogenase, C-reactive protein), kidney biopsy (at the judgment of the attending nephrologist), and treatment modalities (at the discretion of the nephrologist in charge). Notably, systemic atherosclerosis was assessed by examining the clinical involvement across four vascular territories—cerebral, carotid, coronary, and peripheral. A patient was considered to have systemic atherosclerosis if they had clinically relevant disease in any one of these territories. This included evidence of coronary artery disease, cerebrovascular disease, carotid artery disease, or peripheral artery disease as confirmed by appropriate surgical histories, diagnostic imaging, or clinical measures like an ankle–brachial index of less than 0.9.

Renal impairment etiology was classified as definite cast nephropathy if it was histologically confirmed, and as probable cast nephropathy if the involved chain burden exceeded 500 mg/L, accompanied by predominant light chain proteinuria in cases where a renal biopsy could not be performed [3].

The primary outcome of interest in this study was mortality, which was defined as death from any cause. The aim of the study was to identify factors associated with mortality at the time of diagnosis in patients with multiple myeloma and kidney impairment.

The follow-up data on patients, including information on death and end-stage kidney disease (ESKD) events, were obtained from the Romanian Renal Registry and the National Assurance Database system. These comprehensive data sources allowed us to track the patients’ clinical outcomes during the study period. However, it is important to note that the available data did not include the specific dates of the events. Consequently, while we were able to analyze the incidence of death and ESKD in the patient cohort, the lack of event dates limited our ability to conduct more detailed survival analyses, such as calculating mean or median follow-up time, or performing Kaplan–Meier or Cox regression analysis.

Data accuracy was ensured by having two independent reviewers extract and cross-check the data, with discrepancies resolved through discussion and consensus. The study was approved by the institutional review board (“Dr. Carol Davila” Teaching Hospital of Nephrology, Bucharest, Romania), and patient confidentiality was maintained throughout the study.

### 2.3. Statistical Analysis

Descriptive statistics were used to summarize the characteristics of the study population, including mean or median and interquartile range (IQR) for continuous variables and frequency and percentage for categorical variables.

The primary outcome variable was mortality, which was defined as death from any cause. Mortality rates were calculated as the number of deaths divided by the total number of patients in the study. To identify the factors associated with mortality, univariate binary logistic regression analyses were conducted for each predictor variable. Variables that were significant at the 0.05 level were included in the multivariate analysis. Multivariate binary logistic regression was used to identify independent predictors of mortality after adjusting for potential confounding factors. The results were expressed as odds ratios (ORs) with 95% confidence intervals (CIs).

Given that time-to-event data were not available, additional analyses, such as competing risk regression or restricted mean survival time analysis, were not possible. Therefore, the focus of this study was on identifying predictors of mortality at the time of diagnosis.

All analyses were performed using statistical software (SPSS version 26, Chicago, IL, USA) with a two-tailed *p*-value < 0.05 considered statistically significant.

## 3. Results

### 3.1. Patient Characteristics and Clinical Features at Presentation

A total of 89 consecutive patients with newly diagnosed MM and kidney impairment were identified. The median age was 66 years and 38% were male. The most frequent comorbidities were arterial hypertension (71%), systemic atherosclerosis (58%), ischemic heart disease (44%), and diabetes mellitus (12%) (Table 1).

The most frequent clinical features at presentation were asthenia (75%), followed by bone pain (51%), orthostatic hypotension (14%), peripheral neuropathy (10%), edema (10%), and dyspnea (6%) (Table 1).

### 3.2. Multiple Myeloma Characteristics

**Figure 1:**
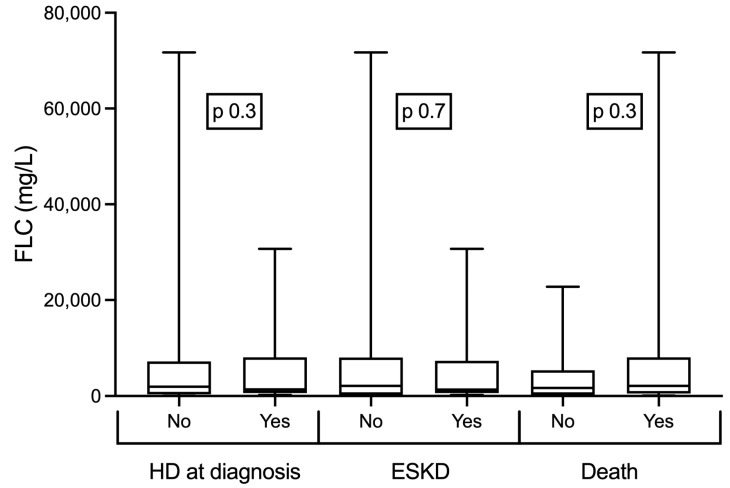
There was no relationship between serum free light chain (FLC) levels and hemodialysis (HD) initiation at diagnosis, end-stage kidney disease (ESKD), and death.

The most common MM type was light-chain-restricted (55%) followed by IgG (30%), IgA (14%), and IgM (1%). Kappa FLC was more frequent than lambda FLC (53 vs. 47%). The median FLC level was 1760 (IQR 450, 7373) mg/L, and 23 (26%) patients had levels of involved FLC under 500 mg/L, and the median marrow plasmacytosis was 30 (IQR 15, 60) % (Table 1). There was no relationship between FLC levels and hemodialysis initiation at diagnosis, ESKD, and mortality (Figure 1).

**Figure 2:**
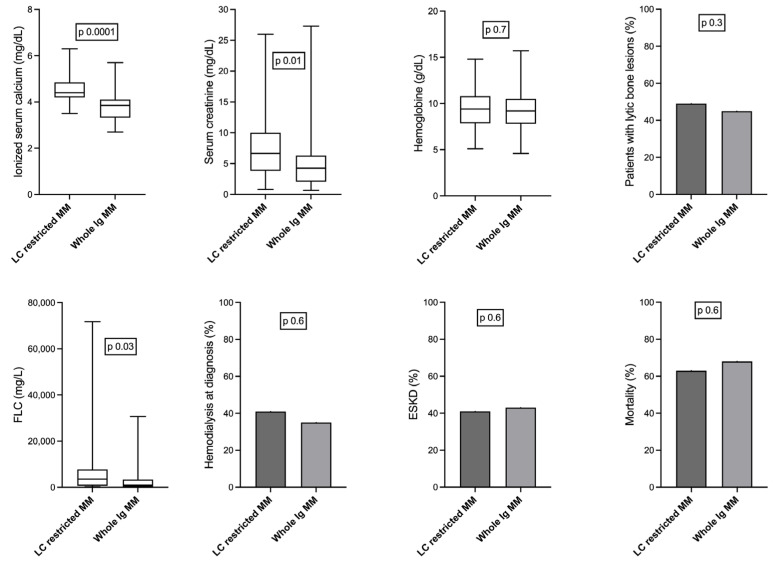
Comparison between patients with light chain (LC)-restricted multiple myeloma (MM) and patients with whole immunoglobulin (Ig) MM; patients with LC-restricted MM had higher ionized serum calcium and serum creatinine, but similar hemoglobin levels and percentage of lytic bone lesions (upper row—“CRAB”); patients with LC-restricted MM had higher levels of free light chains (FLCs), but similar percentages of hemodialysis at initiation, end-stage kidney disease (ESKD), and mortality (lower row).

Patients with light-chain-restricted MM had higher serum creatinine and ionized serum calcium levels and increased levels of FLC compared to those with whole immunoglobulin MM. However, both groups presented with a similar percentage of lytic bone lesions and hemoglobin levels. Also, light-chain-restricted MM was not related to hemodialysis initiation at diagnosis, ESKD, and mortality (Figure 2).

### 3.3. Renal Characteristics

Among the 89 patients, 81% presented with acute kidney injury (AKI), 12% with chronic kidney disease, 5% with isolated proteinuria, and 2% with nephrotic syndrome. Baseline median serum creatinine and eGFR were 5 mg/dL and 9 mL/min, respectively. Median proteinuria was 3.3 (IQR 1.3, 6) g/g with a median albuminuria value of 0.27 (IQR 0.11, 0.66) g/g (Table 1).

Kidney biopsy was performed in 20 (23%) of patients; thus, the etiology of MM-associated kidney impairment was identified as definitive cast nephropathy in 11 (12%) patients, probable cast nephropathy in 51 (57%), amyloidosis in 6 (8%), light chain deposition disease in 3 (3%), and hypercalcemia-related in 15 (17%). In 3 (3%) patients, no likely etiology was identified, as renal biopsy could not be performed due to shrunken kidneys.

### 3.4. Treatment and Outcomes

In our cohort, 34 (38%) patients required hemodialysis at diagnosis, of which 6 (18%) recovered kidney function. However, during the follow-up period, an additional nine patients progressed to ESKD. Consequently, the total number of patients with ESKD in our cohort reached 37 (42%). The mortality rate in the ESKD group was high, with 35 (95%) patients succumbing to the disease.

Treatment with dexamethasone was started by the nephrologist in 74% of the patients at a median dose of 128 (IQR 96, 160) mg, and afterward, all the patients were referred to a hematology service for a bortezomib-based regimen (Table 1). Dexamethasone was administered daily for a period of four consecutive days.

### 3.5. Risk Factors for Mortality

During the study period, 58 (65%) patients died, and they had higher serum creatinine, needed hemodialysis at diagnosis more often, and had lower serum albumin, higher LDH, and increased inflammation (Table 1). In univariate logistic regression, the risk factors at diagnosis associated with mortality were increased age, atherosclerotic burden, low serum albumin, inflammation, increased LDH, and the need for hemodialysis at diagnosis. However, in multivariate analysis, only low serum albumin and the need for hemodialysis at diagnosis were significantly associated with mortality (Table 2).

## 4. Discussion

In the present study, we aimed to investigate from a nephrological perspective the patients with myeloma-related kidney impairment at the time of diagnosis, which is a significant association at the intersection of hematology and nephrology. We report that these patients more frequently have light-chain-restricted MM and most often present with AKI, with approximately one-third requiring hemodialysis at diagnosis, and half progressing to ESKD. Furthermore, hypoalbuminemia and the initiation of hemodialysis at diagnosis were significantly related to increased mortality.

Patients with MM who also suffer from kidney dysfunction typically experience worse outcomes, despite receiving intensive therapy [1,5,6]. Nonetheless, when treated with bortezomib-based chemotherapy, 50–80% of newly diagnosed MM patients have shown considerable improvements in kidney function [7,8]. This includes those who initially required hemodialysis or had serum creatinine levels higher than 5 mg/dL, with post-treatment values often dropping significantly, commonly falling below 2 mg/dL [1,7,8]. Therapeutic recovery of kidney function seems to carry prognostic importance, since patients with MM and AKI who recuperated experienced a notably longer median survival time than those without renal recovery (Table 3) [9,10,11]. However, a cohort study by Gonsalves et al., which included 1135 consecutive patients, revealed that the life expectancy of patients who had reversal of renal impairment still remains inferior compared to those with normal renal function at the time of diagnosis [12].

The percentage of patients with kidney impairment at MM diagnosis who require hemodialysis varies widely, according to the literature, with reported values ranging from 4% to 67% (Table 3). In our study, we found a comparable percentage, with 38% of patients requiring hemodialysis at the time of diagnosis. Despite more than two-thirds of patients receiving dexamethasone upon admission and all patients being immediately referred to a hematology service for a bortezomib-based regimen, the rate of renal recovery remained relatively modest, at only 18%.

Mortality rates for patients with cast nephropathy at diagnosis have been reported to range between 8% and 64% in various studies (Table 3). In our study, we observed a similar high mortality rate of 65% during a follow-up period of 7 years and 6 months.

The increased prevalence of ESKD, the modest response to therapy, and the high mortality rate observed in this study could be attributed to the unique characteristics of the patient population being investigated. The patients in our cohort exhibited more severe kidney impairment than the norm, as evidenced by the median eGFR of 9 mL/min. Moreover, the high percentage of patients requiring hemodialysis at diagnosis also indicates the severity of their condition, which could have contributed to the poor survival rate.

Similarly, Royal et al.’s study on a multicentric population with biopsy-proven light chain cast nephropathy (178 patients, median eGFR 13 mL/min) reported 47% requiring hemodialysis at diagnosis and a 58% mortality rate at the 13-month follow-up [13]. Additionally, Yadev et al.’s investigation of 103 patients with biopsy-proven light chain cast nephropathy (median eGFR 7 mL/min) revealed 67% needing hemodialysis at diagnosis and a 64% mortality rate during follow-up [14].

The findings from these studies suggest that lower eGFR values and the need for hemodialysis at diagnosis are associated with higher mortality rates in patients with myeloma-related renal impairment (Table 3).

**Table 3:** Comparison with similar studies in patients with myeloma-related kidney impairment at diagnosis.

Study/ Year/ Country	Blade et al. [9]/ 1998/ Spain	Knudsen et al. [10]/ 2000/ Denmark	Suyani et al. [15]/ 2011/ Turkey	Dimopoulos et al. [16]/ 2013/ Greece	Park et al. [17]/ 2014/ Korea	Gonsalves et al. [12]/ 2015/ USA	Dimopoulos et al. [18]/ 2017/ Greece	Ho et al. [19]/ 2019/ Australia& New Zealand	Royal et al. [13]/ 2020/ Multicenter: Europe and North America	Yadav et al. [14]/ 2020/ UK	Chen et al. [20]/ 2020/ China	Sharma et al. [21]/ 2022/ India	Present Study/ Romania
No of KI patients	94/423 (22%); SCr >2 mg/dL	225/775 (29%); SCr > 1.47 mg/dL	8/30 (27%); SCr > 2 mg/dL	133 eGFR < 60 mL/min	117/ 379 (31%); eGFR <60 mL/min	192/1135 (20%) eGFR < 40 mL/min	52 (only HD patients included)	383/ 1069 (36%)	178 LCCN KB proven	103 LCCN KB proven	121/393 (31%) eGFR < 40 mL/min	91/216 (42%)	89
eGFR at diagnosis (mL/min)	NA	NA	11	32	NA	CrS 2.6 mg/dL	NA	39	13	7	16.3	15.3	9
Type of FLC involved most frequently	Lambda (51%)	Kappa (31%)	Kappa (88%)	NA	NA	NA	NA	Kappa (63%)	Lambda (53%)	Kappa (54%)	NA	Kappa (61%)	Kappa (53%)
Involved FLC median (mg/L)	NA	NA	NA	NA	NA	2100	9080	NA	5010	7531	NA	NA	1760
LC-restricted MM in KI (%)	32	42	38	21	NA	32	NA	25	48	48	30	26	55
HD required at diagnosis (%)	36	4	50	8	15	16	6 (52 of 796)	4	47	67	17	17	38
Factors associated with improved survival	SCr < 4 mg/dl; response to chemotherapy	Younger age; Stage I, II MM; hypocalcemia; renal recovery	Renal recovery	NA	Complete renal response	Age < 70 y; HD free; absence of high-risk cytogenetics	HD free; absence of high-risk cytogenetics	No KI	Higher eGFR; hematological response (VGPR or higher)	HD free under 12 mo from diagnosis	No KI	HD free; hematologic response	No HD at diagnosis; higher serum albumin
Mortality in KI (%)	29 (first 2 months)	12 (first 3 months)	13 (first 3 months)	8 (first 2 months)	NA	16 (first 6 months)	16	31	58 (at a median of 13 months after diagnosis)	64	23 (first 2 months)	15 (first 2 months)	42 (during the study period)

eGFR, estimated glomerular filtration rate; FLC, free light chain; HD, hemodialysis; KB, kidney biopsy; KI, kidney impairment; LCCN, light chain cast nephropathy; MM, multiple myeloma; NA, not assessed; SCr, serum creatinine.

In multiple studies, light-chain-restricted MM patients have been shown to be more susceptible to kidney impairment [9,10,13,14,15,16] and exhibit poorer kidney survival (Table 3) [10,12]. Our study presents the largest proportion of light-chain-restricted MM patients with kidney impairment to date. Although these patients demonstrated higher serum creatinine and increased serum calcium levels compared to the whole Ig MM patients, we found no significant differences in kidney and overall survival between the two groups.

In our study, we identified hypoalbuminemia as a risk factor for survival in patients with MM. This finding underscores the importance of monitoring serum albumin levels as a valuable prognostic indicator, which can help guide treatment decisions and optimize patient management [22].

This association can be attributed to several interconnected factors, including inflammatory response, nutritional status, disease burden, and kidney dysfunction. MM often induces a systemic inflammatory response due to the uncontrolled proliferation of plasma cells. This response results in increased cytokine production, including interleukin-6 (IL-6), which is a potent myeloma cell growth factor reflecting disease severity and cell proliferation [23,24]. Elevated IL-6 levels can impair the liver’s ability to synthesize albumin, leading to hypoalbuminemia [25]. Hypoalbuminemia may also indicate malnutrition, a common issue in oncologic patients. Malnutrition can weaken the immune system, impair treatment response, and heighten the risk of complications, ultimately contributing to worse survival outcomes [26,27]. Furthermore, IL-6 can have a catabolic effect, promoting muscle wasting and inhibiting albumin synthesis, exacerbating malnutrition in MM patients [27,28]. Moreover, a higher tumor burden can increase monoclonal protein production, displacing albumin in the bloodstream and often correlating with more aggressive disease and poorer prognosis [25,27]. This relationship is the rationale behind incorporating serum albumin levels into various staging systems, such as the International Staging System (ISS) and Southwest Oncology Group (SWOG) criteria [29,30]. Finally, kidney dysfunction in MM may result in proteinuria, causing a subsequent loss of albumin in the urine with an increased risk of complications and mortality as kidney function worsens.

The first admission of MM patients with renal impairment offers a crucial opportunity for early intervention and management. It is imperative to assess the extent to which kidney impairment at diagnosis influences patient outcomes, particularly in the context of survival rates. In line with this, our research highlights the necessity for individualized management strategies for multiple myeloma patients with kidney impairment at the time of diagnosis.

It is important to consider the limitations of our study on myeloma-related kidney impairment at diagnosis. First, the study’s retrospective design might have introduced selection bias and limited the generalizability of our findings. Second, being a single-center study, the results may not be applicable to other centers with different patient populations or practice patterns. Third, the study only includes data from the first admission in nephrology, which may not capture the full clinical trajectory of the patients (for example, autologous stem cell transplantation).

Despite these limitations, our study has several strengths. It is the largest Eastern European study on the subject, which adds valuable insights to the existing body of knowledge. Additionally, the use of hard endpoints obtained from comprehensive national registries lends credibility to our findings and reinforces the study’s rigor.

## 5. Conclusions

In conclusion, patients with myeloma-related kidney impairment who present to the nephrologist more frequently exhibit light chain restriction and most often present with AKI, with one-third requiring hemodialysis at diagnosis. Moreover, hypoalbuminemia and the initiation of hemodialysis at diagnosis were significantly associated with increased mortality. These findings emphasize the importance of interdisciplinary care involving both nephrology and hematology specialists to optimize treatment strategies and improve patient outcomes in this challenging patient population.

## Figures and Tables

**Table 1:** Patient characteristics at diagnosis and comparison between survivors and non-survivors.

	Total N = 89	Survivors n = 31	Non-Survivors n = 58	*p*
Age (years)	66 (60–74)	62 (52–66)	70 (63–78)	<0.001
Male sex (%)	38	45	35	0.3
Comorbidities (%)				
Arterial hypertension	71	71	71	0.9
Systemic atherosclerosis	58	32	72	<0.001
Ischemic heart disease	44	26	53	0.01
Diabetes mellitus	12	7	16	0.2
Clinical presentation (%)				
Bone pain	51	55	48	0.5
Asthenia	75	58	85	<0.001
Dyspnea	6	3	7	0.4
Edema	10	10	10	0.9
Orthostatic hypotension	14	10	16	0.4
Peripheral neuropathy	10	13	9	0.5
Type of MM (%)				0.2
IgG kappa	21	16	24	
IgG lambda	9	10	9	
IgA kappa	6	7	5	
IgA lambda	8	7	9	
IgM lambda	1	3	0	
Free kappa	26	39	35	
Free lambda	29	18	18	
FLC type (%)				0.2
Kappa	53	61	48	
Lambda	47	39	52	
Kappa to lambda ratio	5.6 (0.03–271.33)	31.5 (0.06–380.95)	0.38 (0.01–108.90)	0.1
FLC level (mg/L)	1760 (450–7373)	1583 (277–5134)	1963 (514–8000)	0.3
Marrow plasmacytosis (%)	30 (15–60)	38 (15–69)	30 (15–54)	0.3
Lytic bone lesions (%)	47	58	41	0.1
Hemoglobin (g/dL)	9.4 (7.8–10.6)	9.6 (7.7–11.5)	9.3 (8.1–10.4)	0.2
M-component presence (%)	61	54	65	0.3
M-component level (%)	36.0 (18.5–49.0)	38 (26–51)	36 (18–49)	0.6
Total serum protein (g/dL)	7.4 (6.7–8.4)	7.7 (7.1–9.0)	7.3 (6.6–8.3)	0.08
Serum albumin (g/dL)	3.8 (3.3–4.3)	4.4 (3.7–4.6)	3.7 (3.1–4.0)	<0.001
Total serum calcium (mg/dL)	9.9 (9.1–10.5)	9.9 (9.5–10.6)	9.7 (8.9–10.3)	0.1
Ionic serum calcium (mg/dL)	4.2 (3.8–4.5)	4.2 (3.9–4.4)	4.1 (3.7–4.7)	0.8
LDH (UI/L)	219 (186–315)	201 (180–230)	236 (200–334)	0.01
C-reactive protein (mg/L)	9 (3–27)	3 (1–17)	14 (5–44)	<0.001
Nephrotic syndrome (%)				0.2
AKI	81	71	86	
CKD	12	16	10	
Nephrotic syndrome	2	3	2	
Isolated proteinuria	5	10	2	
Serum creatinine (mg/dL)	5.0 (2.9–8.2)	2.5 (1.35–8.18)	5.80 (3.85–9.60)	0.02
Proteinuria (g/g)	3.3 (1.3–6.0)	3.0 (1.0–6.0)	3.6 (1.4–6.0)	0.3
Albuminuria (g/g)	0.27 (0.11–0.66)	0.24 (0.09–0.46)	0.29 (0.13–0.75)	0.3
Alb to prot-uria ratio <10% (%)	55	61	52	0.4
Bence Jones protein (%)	54	55	54	0.9
Dexamethasone treatment (%)	74	71	76	0.6
Dexamethasone dose (mg)	128 (96–160)	128 (112–160)	128 (80–160)	0.2
Hemodialysis initiation at diagnosis (%)	38	19	48	<0.01
Kidney survival (%)	58	93	40	0.01

M-component determined in serum; Alb, albumin; AKI, acute kidney injury; CKD, chronic kidney disease; FLC, free light chain level; Ig, immunoglobulin; LDH, lactate dehydrogenase; M, monoclonal; MM, multiple myeloma.

**Table 2:** Factors associated with mortality in univariate and multivariate binary logistic regression (i.e., death as dependent variable).

	Univariate		Multivariate	
	OR (95% CI)	*p*	OR (95% CI)	*p*
Age (years)	1.07 (1.03, 1.12)	0.001	1.07 (0.98, 1.16)	0.09
Male sex	1.56 (0.64, 3.81)	0.3	-	
Comorbidities vs. absence				
Systemic atherosclerosis	5.51 (2.13, 14.22)	<0.001	0.11 (0.01, 1.16)	0.06
Ischemic heart disease	3.30 (1.26, 8.58)	<0.01	1.48 (0.18, 11.74)	0.7
Clinical presentation				
Asthenia vs. absence	3.93 (1.43, 10.76)	0.01	0.22 (0.03, 1.35)	0.1
FLC type: kappa vs. lambda	0.58 (0.24, 1.43)	0.2	-	
M-component (%)	0.99 (0.96, 1.02)	0.9	-	
LC restricted MM vs. whole Ig MM	0.82 (0.34, 2.00)	0.6	-	
Serum albumin (g/dL)	0.28 (0.13, 0.63)	0.01	0.22 (0.05, 0.90)	0.03
Total serum calcium (mg/dL)	0.94 (0.69, 1.28)	0.9	-	
C-reactive protein (mg/L)	1.05 (1.01, 1.09)	0.01	1.02 (0.98, 1.06)	0.2
LDH (UI/L)	1.01 (1.00, 1.02)	0.01	1.00 (0.99, 1.01)	0.1
Serum creatinine (mg/dL)	1.06 (0.96, 1.16)	0.2	-	
Alb to prot-uria ratio <10%	0.69 (0.27, 1.77)	0.4	-	
Bence Jones protein vs. absence	0.96 (0.34, 2.72)	0.9	-	
Dexamethasone treatment vs. absence	1.28 (0.48, 3.43)	0.6	-	
Dexamethasone dose (mg)	0.99 (0.98, 1.00)	0.2	-	
HD initiation at diagnosis vs. absence	2.94 (1.04, 8.26)	0.04	10.64 (1.71, 66.14)	0.01

Alb, albumin; FLC, free light chain; HD, hemodialysis; Ig, immunoglobulin; LC, light chain; LDH, lactate dehydrogenase; M, monoclonal; OR, odds ratio.

## Data Availability

The data underlying this article will be shared on reasonable request by the corresponding author.
